# Framing Public vs Private Violence: An Inductive Thematic Analysis of The Australian, The Sydney Morning Herald, the Australian Broadcasting Corporation, and The West Australian

**DOI:** 10.1177/10778012241309367

**Published:** 2025-01-02

**Authors:** Jade McGarry

**Affiliations:** 1 5723 Griffith University, Brisbane, Australia

**Keywords:** domestic violence, terrorism, security, journalism, Australian media

## Abstract

In Australia, *domestic and family violence* (DFV) has reached epidemic proportions. This research argues that it constitutes a form of *terrorism*, although the news media, governments, or public rarely refer to DFV in this way. This paper examines how Australian news media outlets—*The Australian,* the *Australian Broadcasting Corporation*, *The Sydney Morning Herald* and *The West Australian—*reported on and at times connected DFV and terrorism, finding that DFV and terrorism were connected in several ways, and that DFV was described as terrorism by several academics, advocates, journalists, and victims. The research also found that the framing of each issue differed, subsequently affecting public perceptions of the perceived severity, impact, and pervasiveness of each issue.

Since the turn of the 21st century, *terrorism* has been central to the public sense of security and insecurity (Neal, 2009). The term has rightly been used in reference to acts of violence carried out with an ideological, political, or religious motivation, with the intent to coerce governments and people more generally (Law, 2016). Although it is the case that the public has witnessed horrific acts of political terrorism over the last two decades that have resulted in thousands of deaths, this is overshadowed by the level of fear, violence, and death resulting from *domestic and family violence* (DFV). Significant literature and victims, perpetrators, and advocates have proposed the term terrorism in reference to DFV (Edwards, 2015; Esman, 2020; Hill, 2019; Johann, 1994; Johnson & Leone, 2005; Marcus, 1994; McCulloch et al., 2019; Pain, 2014; Perilla, 1999, Sloan-Lynch, 2012), focusing on the combination of power, coercion, and control at the heart of this form of violence and abuse, alongside the imposition of structural power that inherently disadvantages victims of DFV. However, the media and political and public discourses have shown a reluctance to use the term terrorism in reference to DFV.

This paper examines terrorism regarding the (in)security threat presented by the DFV issue in Australia. This research draws upon Buzan's (1991) definition of security as “the pursuit of freedom from threats” (p.18), specifically protection (or lack thereof) from crime, danger, fear, or anxiety. The purpose of this is to examine and understand “how” and “why” we use certain terminology in relation to certain phenomena, how these impact on our sense of (in)security, and how we as a society respond to the phenomena. The research employs the primary methodology of inductive thematic analysis (Naeem et al., 2023) and other manifest units of data to gain a comprehensive understanding of the way DFV and terrorism are presented in Australian news media. *The Australian, The Sydney Morning Herald,* the *Australian Broadcasting Corporation* (ABC)*,* and *The West Australian* are analyzed regarding their coverage of both DFV and terrorism over a 10-year period between 2013 and 2022. The purpose of this is to understand the way the stories were framed, the type of language that was used, and to identify the emerging trends and patterns of the coverage that affect the way audiences understand and grade the severity and nature of both issues. This research aims to reframe the way audiences understand DFV through experimenting with language, harnessing the socio-political influence and impact of the term terrorism, and to address and expand upon the work being done to reduce the epidemic of DFV in Australia.

## Domestic and Family Violence in Australia

In Australia, one woman is killed each week by their current or former partner and one in four women have experienced physical or sexual violence by a current or former partner since the age of 15 years (Our Watch, 2024a). DFV is the leading cause of youth homelessness (Meyer et al., 2023), and abuse and neglect during childhood have consistently been shown to be the leading behavioral risk factors contributing to the burden of suicide and self-harm (Australian Institute of Health and Welfare [AIHW], 2021). In Western Australia in 2017, half of women and children who died by suicide suffered from DFV (Ombudsman Western Australia, 2022). The DFV issue in Australia is of significant concern (AIHW, 2023) and has been described as a national crisis that has reached “epidemic proportions” (Australian Government Department of Social Services, 2023).

Although DFV is certainly not a new phenomenon, the term “domestic violence” has changed shape and shifted over time (Alexander, 1993). Terms such as “wife-battering” or “wife basher” were previously used to describe violence occurring between intimate partners. Language such as this proves reductive as it disregards many of the other forms of abuse that take place in DFV situations including emotional abuse, coercive control, financial abuse, child abuse, and sexual abuse (Full Stop Australia, 2024). This research focuses, in a large part, on this shift in language, and subsequently on public perceptions of the issue. Words have power (Conley et al., 2019) and, as this paper explores, the dramatic shift of DFV from the dark shadows of private family life to the light of public conversation, law, and policy is one that began with a shift in understanding how we define or describe a phenomenon, and why that definition or description matters.

In Australia, DFV is defined by the *Family Law Act 1975* as “violent, threatening or other behaviour by a person that coerces or controls a member of the person's family or causes the family member to be fearful” (Commonwealth of Australia, 1975: section 4AB). Australian national leader in the prevention of violence against women and their children, Our Watch (2024b) defines DFV as “acts of violence that occur in domestic settings between two people who are, or were, in an intimate relationship, including physical, sexual, emotional, psychological and financial abuse” (para. 4).

## Theorizing Terrorism

Terrorism has been labeled the “scourge of our age” (Smith, 2019, p. 1), certainly coming to the forefront of the agenda of international relations and other academic disciplines as a direct result of September 11, 2001, attacks (Wight, 2009). The Australian Government has described terrorism as a “dynamic threat” (Australian Government, 2010; 2017). This dynamism provides space for reflective, honest, and conceptual academic investigation of terrorism as a phenomenon, as well as a technique and tool used by individuals in the pursuit of power and control.

Researchers working within terrorism studies have attempted to understand, define, and address the complex phenomena of terrorism (Borum, 2010; Crenshaw, 2010; Hoffman, 2017; Kydd & Walter, 2006; Martin, 2017; Moghaddam & Marsella, 2004; Poland, 1988; Saul, 2006; White & Clear, 2003), although there is no consensus within this literature about the *nature* of this form of violence and resistance (De la Calle & Sánchez-Cuenca, 2011). Terrorism studies itself has been described as an increasingly important subfield within many disciplines, with the literature arguing that the rapid growth in studies of terrorism “enhances the significance of identifying works that make substantial conceptual and empirical contributions” (Lentini, 2008, p. 133). Although it is crucial to note that the discipline has received significant criticism, being described as “too narrow, too event driven and too strongly tied to governments’ counterterrorism studies” (Schuurman, 2019, p.463). Walter Reich’s (1998) *Origins of Terrorism: Psychologies, Ideologies, Theologies, States of Mind* laments this literary disconnect and confusion, arguing that,

Terrorism is so varied and complex a phenomenon that it should give pause to anyone whose aim it is to understand it – or, to be more precise, whose aim it is to understand the many different terrorisms that the deceptively singular term covers (p. 262).

The word “terrorism” originates from the Latin word *terror*, described as a great fear or dread, the term is directly related to the Latin verb *terrere* (to frighten). The ancient Romans first used the term *terror cimbricus* to describe the fear and panic that was felt in preparation of an attack by the fierce warrior Cimbri tribe in 113 BC (Escareno, 2013). The French later used the term during the Reign of Terror (1793–94), when the government enforced severe punishments (often death) upon those who were believed to be against the continuation of the French Revolution (Erlenbusch, 2015; Palmer and Woloch, 2013). The first English definition of terrorism was “a state of being terrified, or a state of impressing terror” (Merriam-Webster, 2023). The US *Code of Federal Regulations* now defines terrorism as “the unlawful use of force and violence against persons or property to intimidate or coerce a government, the civilian population, or any segment thereof, in furtherance of political or social objectives” (28 Code of Federal Regulations, Section 0.85).

It is evident that the definition of the term has been heavily contested in academia, government, and by the public (Greene, 2017; Meisels, 2009; Schmid, 2023), but a consistent focus on terrorists’ use of fear has been a common thread found across definitions (Esmailzadeh, 2023; Garrison, 2004; Richards, 2018; Saul, 2006). For example, Healey (2011) found that “terrorism is the use or threat of violence to create a climate of fear in a given population” (p. 1), with further research finding this fear is then used to manipulate and control the victim (Altheide, 2006). The term terrorism, both socially and culturally, has a significant amount of power and influence, in particular over the functioning of democracy and public perceptions of (in)security. Chomsky and Herman's (1979) seminal *The political economy of human rights,* speaks explicitly to this power and influence, finding that identifying or labeling an event as ‘terrorism’ “can increase the importance assigned to an incident” and “allow greater freedom of action by the state” whereas not utilizing this “terrorist label” can “diminish the severity of such attacks and limit judicial action” (p.7).

## Domestic and Family Violence as a Form of Terrorism

A considerable number of researchers, DFV advocates, experts and victim-survivors have explored the idea of describing DFV as a form of terrorism (Anderson, 2009; Edwards, 2015; Hayes & Jeffries, 2016; Johnson et al., 2014; McCulloch et al., 2019; Silverzweig, 2010; Smith, 2019; Straus & Gozjolko, 2014; Walker-Munro & Walker-Munro, 2023; Zimmermann, 2018), finding that the framework of understanding DFV as terrorism is essentially twofold: political and nonpolitical.

The first approach (nonpolitical) is stripping it (terrorism) of its politicized historical context (that being directly related to current perceptions of terrorism as inherently related to state strategization and international politics [Harris-Hogan et al., 2016]) exploring the notion of power, control, and fear used by the perpetrator as well as the terror experienced by the victim. At the crux of this theoretical approach lies coercive control. Feminist researchers, Hayes and Jeffries (2016) speak to this notion of coercive control in relation to DFV in what they describe as *romantic terrorism,* finding that DFV perpetrators are terrorists who operate in Australia every day, relying on fear and intimidation to control their victims. The authors argue that due to the intimacy of this violence (being domestic and largely hidden from public sight) “it rarely evokes outrage and has never resulted in governments or anyone else declaring a “war” against it” (p. 39). This sentiment speaks to this idea of nonpolitical terrorism that utilizes fear and power to control a victim, and with the protection of privacy, intimacy and “romance,” this type of terrorism is often condoned and not treated with the urgency and action it so desperately requires.

The other theoretical approach (political) is to harness this “politicization.” A key challenge that is often posed regarding this idea of DFV being a form of terrorism is the perceived inherent disconnect between the two issues due to the political nature of terrorism (and DFV lacking this political nature). This research argues that this sentiment is misunderstood. Researchers have found that women who are victims of DFV are pushed into systems that inherently disadvantage them (Brown, 2012; Decker et al., 2019; Robinson & Yoshida, 2022), for example, the courts, the police, and welfare systems. The imposition of structural power (Allen, 2005) disadvantages women and ultimately prevents them from engaging in society in a meaningful way. Sloan-Lynch's (2012) *Domestic abuse as terrorism* speaks to this notion finding that DFV is:

A commonly overlooked form of domestic terrorism that coerces women into behaving in clearly detrimental ways and accepting disadvantageous social arrangements by means of institutionalized methods of violence and terror creation (p. 775).

## Media Framing of Violence

What and how the media reports has social and political impacts on intercommunity and social relations (Powell, 2018). Researchers have argued that the purpose of journalism is to describe society to itself (Bennett 2005); therefore, how well the job is being done by journalists and news outlets in Australia is incredibly important because it has the potential to affect the way people understand the nature of these acts of violence, as well as understand the people at the center of these stories (Bleich et al., 2018). The seminal work on *framing* by Entman (1993) explored the power of communicating a text through selection and salience: He argued that “to frame is to select some aspects of a perceived reality and make them more salient in a communicating text, in such a way as to promote a particular problem definition, causal interpretation, moral evaluation, and/or treatment recommendation for the item described” (p. 52). This research draws upon the work of Entman to explore the way Australian media frames (in)security, through analyzing how DFV and terrorism are contextualized and presented to audiences.

The news media's role as the “fourth estate” means it is responsible for covering news stories that assist in the proper functioning of democracy by sustaining an informed populace (McNair, 2009). The media and journalists have an ethical responsibility when covering stories, in particular stories pertaining to violence, to provide balanced, fair reporting, consistent, and vigilant fact-checking, and to ensure that the voices of those affected by an issue are heard throughout the news stories (McGarry et al., 2023). Research has found that, in relation to media coverage of DFV, it has the potential to “provide significant influence on the public's opinion of IPV (intimate partner violence) and related policies that directly impact victims, survivors, and perpetrators” (Kelly & Payton, 2019, p. 48). Significant research has explored Australian media coverage of DFV (Gilmore 2019; Hawley et al., 2018; Karageorgos et al., 2023; Morgan & Politoff, 2012; Simons & Morgan, 2018; Sutherland et al., 2016; Uibu, 2023) finding key issues regarding a lack of contextualization, stereotyping, and an inability (or refusal) to articulate the severity of the issue.

It is vital to draw upon the concept of mediated reality within this discussion, to distinguish between reality itself and the mediated construction of reality or the reality that is presented by the news media and journalists (Couldry & Hepp, 2018). In this research, reality is of particular interest as audiences’ perceptions of (in)security are shaped through the mediated version of DFV presented in the media. A statement made during prominent Australian DFV victim-survivor and advocate, Rosie Batty's (AO) address to the National Press Club by journalist Julie Hare, when she said, “you say that family violence doesn’t discriminate, but in a way media organisations do” (Hare, 2024), spoke directly to media outlets reporting habits. How the news media frames DFV has a significant impact on public perceptions of victims, perpetrators, and the nature and severity of the violence (Gilmore, 2019). The decision to cover some stories rather than others, some victims rather than others, and certain types of DFV rather than others have a significant impact on public perception and awareness of the issue.

## Method

Researchers have found that news media coverage of violence is a central factor in affecting perceptions of crime and increasing levels of fear (Smolej & Kivivuori, 2006). In order to understand how audiences and Australians generally are understanding the nature, severity, and impact of violence, in the case of this research on DFV and terrorism, there must be a focus on how news media covers these acts of violence. This research employs an inductive thematic analysis (Naeem et al., 2023) and other units of data to understand how stories concerning DFV and terrorism are framed, contextualized, and connected.

Significant research has explored media framing of both DFV and terrorism, but this research explores media coverage of both issues together, making it unique and able to address an evident gap in the literature. The purpose of searching for articles that contain both the term “terrorism” and the phrase “domestic violence” is to begin identifying points of connection between these two seemingly disconnected ideas. As previously explained, the literature has begun to identify explicit connections between DFV and terrorism, making a strong case for using the term to describe this form of violence. This research is looking specifically at the Australian news media, which plays a significant role in informing the public and shaping public perceptions of (in)security. The purpose of this is to see if, when, and how this descriptor (terrorism) is ever used, and how the two core security issues (DFV and terrorism) are discussed in conjunction with one another. Several core research questions guide this research:

- What is discussed when “domestic violence” and “terrorism” are both mentioned in a news story?
- What points of connection are made (by the news media) between terrorism and DFV?
- How often is the term “terrorism” used to describe DFV?
- Who uses the term “terrorism” to describe DFV?
- How does coverage differ between stories that are focused primarily on DFV (and mention terrorism in some way) or focused primarily on terrorism (and mention DFV in some way)?
- How is each issue “contextualized” for audiences?

To gain as comprehensive a view as possible of the Australian news media landscape, a variety of news outlets were selected for analysis. Australia's public broadcaster the *Australian Broadcasting Corporation* (ABC), *The Sydney Morning Herald,* owned by Nine Entertainment, *The Australian,* a national newspaper owned by News Corp Australia, and *The West Australian,* owned by Seven West Media, the only locally edited daily newspaper published in Perth, Western Australia. This even distribution of news producers, both ideologically in terms of them being more inclined to be left or right leaning (Muller, 2017), and distribution of ownership, from Nine Entertainment to News Corp enabled a comprehensive view of news media in Australia.

**Figure 1.**
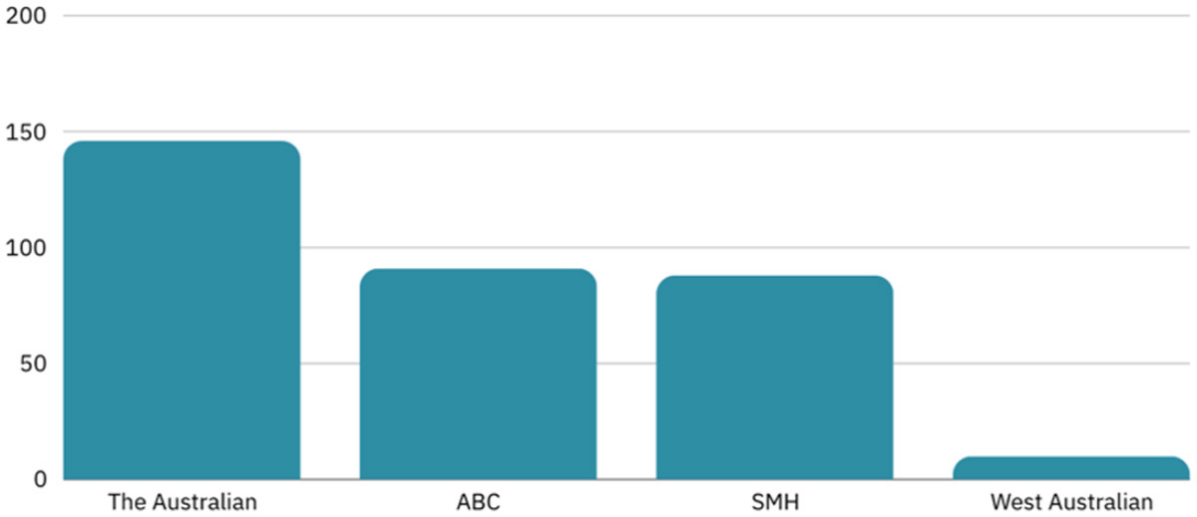
Media coverage of “terrorism” and “domestic violence” by outlet.

The sample of news reports for this study was collected through a search of the Dow Jones Factiva for both the phrase “domestic violence” and the term “terrorism” found anywhere in the article, published between January 1, 2013, and December 31, 2022. The search returned 335 articles: 146 were published by *The Australian*, 91 were published by the ABC, 88 were published by *The Sydney Morning Herald,* and 10 were published by *The West Australian* (see Figure 1).

A coding system (McGarry, 2024) was then developed to assess each article, determining dominant themes, language, the inclusion of DFV data, access to a support service, and identification of the two concepts being discussed together to explore media influence on public perceptions of these forms of violence. This process included the use of manifest analysis (Gray & Densten, 1998) and reflective thematic analysis (Braun et al., 2023).

## Findings

### Contextualizing the Australian Landscape

**Figure 2.**
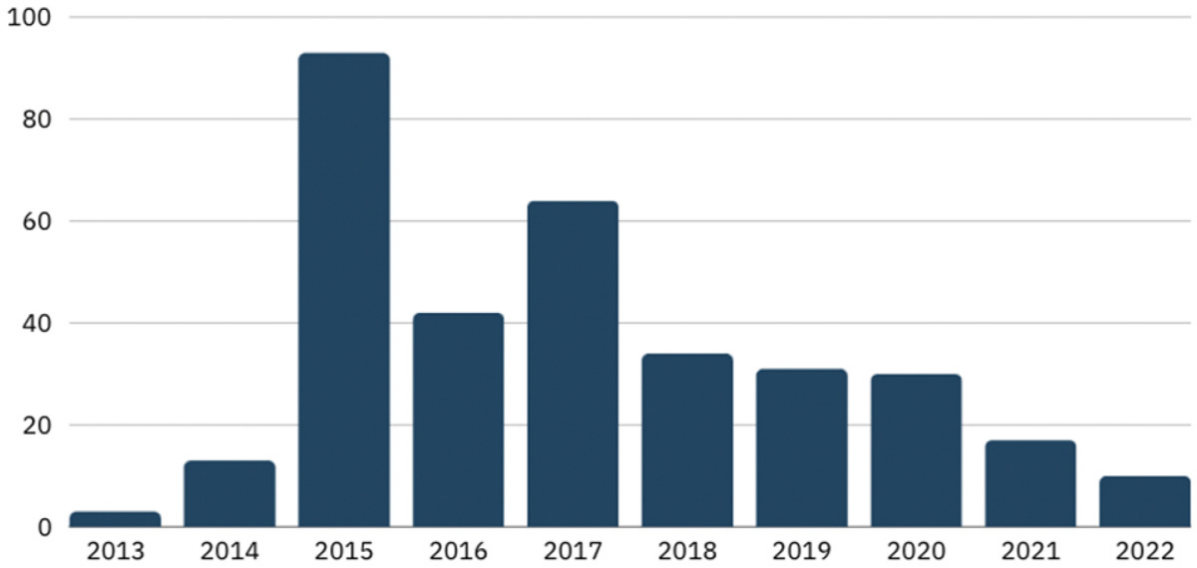
Media coverage of “terrorism” and “domestic violence” by year.

A key finding from this data set was the significant spike in coverage during 2015 (see Figure 2). The influx in news coverage concerning both forms of violence was a result of several key incidents during 2014 and 2015.

Although 2015 showed a significant spike, the events that occurred in 2014 sparked conversations that continued into 2015. First was the death of 11-year-old Luke Batty in Australia on February 12, 2014 (Davey, 2014). Luke was struck on the head and stabbed to death by his father Greg Anderson at cricket practice on a sports oval in the outer Melbourne suburb of Tyabb. The violent incident took place while parents and children were still in the vicinity. This event was a key DFV incident that received considerable news coverage, in particular in the wake of the murder because Luke's mother Rosie campaigned against DFV in Australia (Hawley et al., 2018).

During December 15–16, 2014, the infamous Lindt Café siege in Sydney (Doherty et al., 2014) was framed by Australian news media as a predominately Islamist terrorist-related incident (although the perpetrator did suffer from severe mental illness and a significant history of criminality and violence un-related to terrorism) (Ali and Khattab, 2018) that sparked nationwide coverage and debate (McLinden & Barclay, 2018). The siege led to a 16-hr standoff that resulted in the deaths of three people (including the perpetrator). The event was broadcast across the country and proved to be a monumental event that altered perceptions of the threat of terrorism and perceptions of (in)security.

During January 7–10, 2015, beginning with the shooting attack at Charlie Hebdo offices, a French satirical weekly magazine, then a Jewish supermarket, terrorists killed 14 (BBC, 2015), which again thrust the issue of terrorism into the national and international debate. Following this, in September 2015, a volatile environment was created when the Australian Government approved the re-settlement of 12,000 Syrian refugees (Spinks, 2015) in the wake of several key terrorist incidents across the globe, with key right-wing commentators expressing concern about the perceived connection between Muslim immigration and terrorism (McGarry, 2024). This is explored further later in the paper, but it is useful to keep in mind to contextualize the conversation.

In September 2015, Malcolm Turnbull was elected Prime Minister and pushed a lengthy agenda concerning the plan to “keep jailed terrorists locked up” if they remain a danger to the community after they have completed their sentences (Smith & Nolan, 2016). Turnbull was the leader of the centre-right *Liberal Party of Australia* and his political agenda was orchestrated in tandem with a plan to strip citizenship of foreign fighters (Barker, 2015) showcasing the level of concern that the Australian Government and its people gave to this form of violence. Serious action was being taken. At this time, the public discussion concerning the “growing threat of terrorism” was most certainly at a high. On October 2, 2015, 15-year-old Farhad Jabar had shot and killed unarmed police civilian finance worker Curtis Cheng (Dutton, 2018). The New South Wales (NSW) Police Commissioner, Andrew Scippione, described the event as a “politically motivated act of terrorism” (Lee, 2016).

Meanwhile, in the same year, Rosie Batty (AO) was awarded the prestigious Australian of the Year award (Australian of the Year Awards, 2015) for her DFV advocacy work. This received significant media coverage and Ms Batty was able to give speeches that assisted in furthering her mission, which subsequently resulted in another spike in coverage, in particular in relation to her description of DFV as terrorism.

### Key Themes

**Figure 3.**
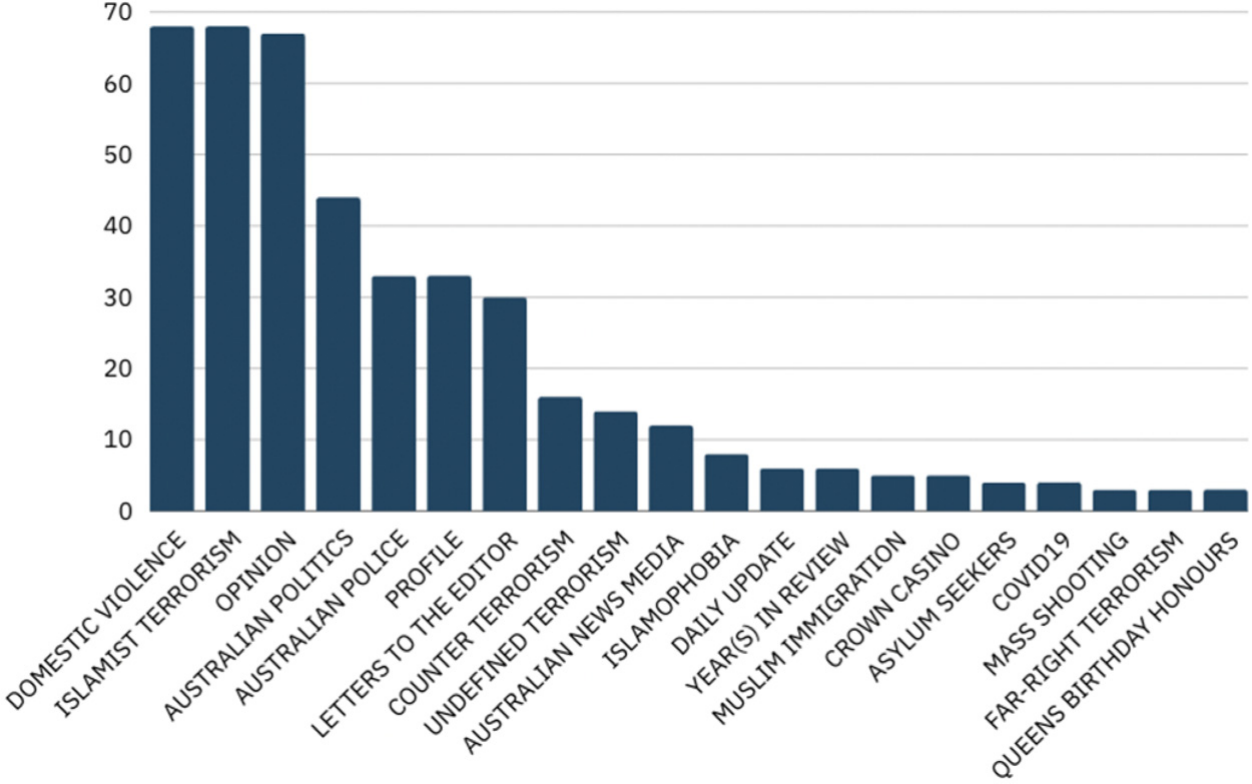
Media coverage of “terrorism” and “domestic violence” by themes.

The purpose of identifying the themes of each article was to understand what exactly is being discussed when the terms “terrorism” and the phrase “domestic violence” are used. The purpose of this is to understand how audiences are understanding the issues both in relation to one another and in relation to other issues. Several dominant themes emerged: Domestic violence, Islamist terrorism, Opinion, Australian politics, Australian police, Profiles, and Letters to the editor, alongside several less re-occurring themes (see Figure 3).

Identifying the themes enabled an assessment of the contexts in which these conversations or news coverage is taking place often subsequently affecting the way in which the story is framed. This form of analysis was able to interpret patterns and themes within the data set, leading to new insights and understandings (Naeem et al., 2023).

#### Theme 1: Domestic Violence

The first and most dominant theme that emerged was “domestic violence” (21%). A key subject under this theme was Rosie Batty and her description of DFV as “intimate terrorism” during her advocacy campaign in the wake of her son's 2014 murder (21% of the articles under the theme of domestic violence). The work of Batty encouraged a nationwide debate about DFV that included support and backlash, particularly regarding language. A 2015 ABC Radio National (RN) transcript titled “We don’t see the terrorism in our own homes: Rosie Batty” included an interview with Batty where she reflected upon the use of the term terrorism to describe DFV.

I think it puts it [DFV] in a context actually that is very uncomfortable and is very confronting…. We take it [terrorism] so seriously that it's amazing the steps governments will go to with the threat of terrorism. But when you define terrorism and the actions, you know, you can closely relate it to what's happening for some families who are experiencing domestic violence and you can very clearly see that it is acts of terrorism within their own family homes. (Batty, 2015)

Domestic Violence also emerges as a theme when it is mentioned as a “previous offense” of terrorists, speaking to an escalation of violence (Smith, 2019). This draws our attention to a distinct and evident lack of media coverage of DFV until what is seen as the worst-case scenario, that being a terrorist incident takes place. This was specifically the case regarding Man Haron Monis, the perpetrator of the infamous Lindt Café siege in Sydney. In the wake of the siege, it was unveiled that Monis had a significant history of DFV (Davey, 2014). A 2015 article titled “Time for Tony Abbott to step up and tackle domestic violence in Australia” published by *The Sydney Morning Herald,* lamented this history and the subsequent public backlash:

He [Monis] had been charged with being an accessory to the murder of his wife, and he faced counts of aggravated sexual assault on other women. Many questioned why this was not enough to have him locked up on remand in the first place. (Metcalfe, 2015)

Another 2017 article titled “Lessons from the Lindt Siege” published by *The Australian* also showed concern:

Serious concerns police had expressed about how Monis came to be granted bail over charges related to the murder of his ex-wife were also not recorded. Barnes expresses concerns about a system that could allow Monis to have been charged in the past for sex offences by serving of a court attendance notice rather than his arrest. (The Australian, 2017)

The research also found a significant number of articles that described the experiences of DFV victims within this theme, these first-hand accounts described the acts violence inflicted upon them as terrorism or being terrorized. In a 2014 ABC RN transcript titled “Vic pollies asked to combat intimate terrorism” a victim described her experience:

Every day you're on edge just in case he's going to come home in a bad mood, just in case out of nowhere something triggers him off and off he goes, so you never feel safe. And I think when you're a mother too and you're trying to protect children from what's going on in the house around you, you're doubly on edge … I was punched, I was kicked, I was threatened to be run over with a car, I was threatened with a knife. (ABC, 2014)

Another key issue that was covered significantly within this theme, was the murder of Brisbane mother Hannah Clarke and her three children. On February 19, 2020, Clarke and her three children Aaliyah (6 years), Laianah (4 years), and Trey (3 years) were ambushed by their father Rowan Baxter while they were in the car. Baxter doused the interior of the car in petrol, setting his estranged wife and three children alight, before stabbing himself to death. This coverage was largely focused on “analysis” or a “breakdown of the events” with key witnesses, family members, and friends quoted and interviewed. It was noted earlier in this article that the severity and brutality of a DFV incident affect the level of coverage, and the fact that this incident took place in public, on a suburban street, evidently caught the media and audience's attention. The nature of media coverage of DFV, that it is case based and intrinsically tied to the courts, means that only cases that reach extremes are covered, meaning that is the only time when audiences often hear about DFV. In the case of Hannah Clarke, the coverage also created space for a nationwide conversation about coercive control. This led to the next emerging section of this theme, the significant coverage of coercive control laws, in particular information regarding the implementation and passing of the laws. The emergence of the theme of “Domestic Violence” signals an evident focus on this issue specifically, and although is to be expected when searching for articles containing both the term terrorism and the phrase domestic violence, it is important to explore exactly what is being discussed. As explained, media coverage of DFV is crucial to shaping public perceptions of the issue, and this research found that DFV was discussed, explicitly, in tandem with the term terrorism. This indicates some level of ‘association, connection and understanding’ between these two concepts, whilst core issues like coercive control, escalation of violence, abuse, first-hand experiences of DFV, intimate partner homicide, filicide (and the impact of these events on family, friends, victims, etc.) are simultaneously being discussed.

#### Theme 2: Islamist Terrorism

The second dominant theme is stories concerning “Islamist terrorism” (20%). A core issue that is covered within this theme of Islamist terrorism is stories pertaining to this notion of “jihad denialism” a term coined by Chris Kenny of *The Australian.* These articles are very reminiscent of a “moral panic” (Cohen, 1972), with Kenny presenting what is akin to a “call to arms,” that “we need to do something about jihad denialism,” and “things have gotten out of control” (Kenny, 2015). This sentiment is often supported using inflammatory language (Akbarzadeh & Smith, 2005) which is often directed toward Islam and is spoken of in conjunction with DFV, specifically regarding Australia's response. For example, in a 2015 article published by *The Australian* titled “Islamist extremism: the menace many dare not speak by name” Kenny stated:

Instead of learning more about Islamist extremism, its origins, aims, methods and the trauma it brings upon Muslim and non-Muslim communities, we are encouraged to avoid the “I” word and treat every terrorist attack as another trigger to embrace all Muslims. This is patronising and muddle-headed. Let us compare it with how we deal with another serious issue confronting our society. In the wake of domestic violence incidents and killings we don’t urge people to embrace all men or stress that not all men should be made to wear the blame. We do quite the opposite. We now urge all men to be aware of this issue, talk about it and do what they can to both prevent it and provide refuge or protection for potential victims. (Kenny, 2015)

This sentiment highlights the frequent inability of media to understand DFV as a social issue, whose origins are rooted in misogyny, patriarchy, and toxic masculinity that shape perceptions of women and the role of women in Australian society. It refuses to acknowledge that to address the issue of DFV, men need to be involved in order to disrupt the social underpinnings of this form of terrorism. Kenny's decision to compare this approach with the Muslim community in regard to terrorism disregards the significant body of literature that articulates the importance of involving the Muslim community in challenging these mainstream narratives of an inherent connection between Islam and terrorism (Aly, 2007; Cherney & Murphy, 2016). Challenging these narratives enables Australia's Muslim communities to flourish and be disconnected from radical Islamist extremism that is unrelated to Islam as a religion (McGarry et al., 2023).

Another 2014 article published by Kenny in *The Australian* titled “Patience of extremist the unpalatable reality*”* presented the same argument regarding responses to Islamist-inspired terrorist attacks:

Consider the inverted sensibility of this now common reaction to praise Islam first, rather than immediately condemn the violence and its evil inspiration. Imagine the outrage if our initial response to murderous domestic violence was to declare that most husbands actually are caring partners. (The Australian, 2014)

This mediated inversion of reality is concerning because it fails to acknowledge the importance of ensuring a separation of Islam and extremist Islamist violence, or ideology. The conflation of Islam with Islamism, particularly by news media, has been found to “contribute to misinformation, pejorative public sentiments about Islam and Muslims, and reinforces extremist propaganda” (McGarry et al., 2023, p. 234). Kenny's decision to disregard the significant response by governments, through public policy, border protection, immigration policy, etc., to Islamist extremism, presents a distorted image of the reality and strips the issue of context.

Another key issue that received significant coverage under the theme of Islamist terrorism was the Lindt Café Siege, specifically pertaining to hostage negotiation. The NSW police used techniques (later found to be inadequate) that were reminiscent of the way DFV hostage negotiations were handled. A 2015 article published by *The Sydney Morning Herald* titled “We need a wider conversation on violence” lamented this junction between DFV and terrorism finding that the police responsible for handling the siege had significant experience in hostage negotiation, although this was almost always in the context of DFV incidents. The article argues that the comparison is “useful up to a point, in highlighting how we have failed, as a nation, to take violence against women seriously, despite the fact one in three women report having experienced it” (Metcalfe, 2015). This level of consideration, care, expertise, and technique (in regard to police procedure) is found to be lacking in critique until the violence moves from the “private sphere” (that being the home) to the “public sphere.” This notion speaks to this idea of a “prioritization of security threats,” where terrorism significantly overshadows DFV and the safety of women, and violence is tolerated in the home (or intimate relationships) but not tolerated when it takes place in public.

A 2016 article published by *The Australian* titled “More reliance on special forces” again lamented the effectiveness of Australia's counter terror stance finding that a series of recent terrorist incidents and their police approach required action and review finding that “the so-called “contain and negotiate” approach used in domestic violence sieges is unlikely to work with Islamist terror, as the Lindt siege experience shows (The Australian, 2016). This is noteworthy again as it speaks to this level of care and consideration in regard to terrorism incidents, that is absent in discussions pertaining to DFV.

It is also noteworthy that this theme of Islamist terrorism was a leading theme within the search parameters (of searching for articles containing both the term “terrorism” and the phrase “domestic violence”), indicating that a search for the term “terrorism” results in content pertaining to Islamist terrorism, despite research finding that domestic far-right terrorism is increasing in Australia (Grant, 2024). Also noteworthy is that 17% of all articles in the data set contained the term *Islam.* Researchers have found that Australians get the majority of information about Islam and Muslims from the mass media (Rane et al., 2014), meaning that the way that the religion is covered has a serious impact on public perceptions of Muslims. Identifying the emergent theme of Islamist terrorism was crucial to understanding how terrorism (which was found to be most often related to Islamist-inspired terrorism) is discussed in conjunction with DFV. The reoccurring discussions pertaining to police and policy response to violence saw this theme connect DFV and terrorism, showcasing results that speak to hostage negotiations, approaches in dealing with core security threats (being DFV and terrorism), and escalations of violence. This is important because much of the discussion taking place within this theme finds treating DFV and terrorism as similar issues, in terms of understanding DFV as a social, gendered issue (much the way that terrorism is often understood as a product of Islam) is preposterous and illogical. This is intriguing because this research argues this approach (understanding DFV as a social issue, not a series of isolated incidents) is vital and DFV needs to be dealt with accordingly and considered within the realm of national security.

#### Theme 3: Opinion

The next emergent theme was “Opinion” or “Editorial” articles (20%). The major players writing opinion articles were *The Australian* (70.5%) followed by *The Sydney Morning Herald* (28%). *The Australian's* domination of this theme is noteworthy. The influence and power of the Murdoch media empire is one that has been focussed on in many areas of communication and media research (Kellner, 2012; Lisners, 2013; Vine, 2022) with the literature articulating the scope and influence of the media empire as extending beyond the world of newspaper into many forms of media including magazine and book publishing, network, and satellite television, extending this influence across the globe (Tucille, 1989).

The organizations’ prominence and influence are incredibly relevant to understanding where public perception and attitude stems from and how power, in terms of media ownership, affects the way news is produced, and content is created. *The Australian's* “neo-conservative approach” to news production (Taylor & Collins, 2012) makes this outlet's framing and agenda setting of interest, in particular its domination of the Opinion theme. As previously mentioned, Chris Kenny of *The Australian* was a major contributor in Opinion stories pertaining mostly to Islamist terrorism. Kenny created what is reminiscent of a moral panic, assessing the dangers of what he coins “jihad denialism.” Again, an overwhelming number of opinion articles focused on Islamist terrorism (43%) compared to DFV (15%).

The core issues associated with opinion or commentary-style journalism found, in this case 21% of all articles, when searching in the previously explained parameters is that they are not technically news, evidently presenting issues. Hallock's (2006) *Editorial and opinion: The dwindling marketplace of ideas in today's news* explored the influence, role, and impact of Opinion or Editorial content finding that “the editorial page is the section of the newspaper that serves as a community's conscience, that sets its priorities, that serves as a community sounding board” (p. 9). We can then ask: What does this mean for the level of concern regarding DFV compared to terrorism? Opinion pieces found within this data set are focused far more on Islamist terrorism, why? And why are we, or the Australian news media, not outraged in the same way about DFV?

Firmstone (2019) found that “journalists who write editorials play a powerful role in constructing political debate in the public sphere” (p. 1). Firmstone argued that it can be problematic that these opinion stories, although certainly labeled as such, still stand among news stories as though they are the same. The question of a code of ethics and journalistic, ethical news production is argued to not be held to the same standard. The difference between presenting news issues in a structured manner, compared to what is often “a rant” in opinion pieces is stark, in particular in the data collected from this research. The presentation of objective information with the intent to inform the populace is lost in the case of opinion articles (McCabe & Heilman, 2007).

Another defining element within this theme of Opinion is that the articles are overwhelmingly written by men. This is an interesting aspect that traverses key feminist issues of representation, voice, and authority over narrative and the subsequent silencing of women. The lack of female voices has a profound effect on the way the issue is presented to audiences (Kitzinger, 2002). According to *Women in Media* (2013*)*, women are “severely under-represented in terms of media coverage as both sources and experts in their field,” finding men accounted for 70% of quoted sources, 66% of quoted experts, and dominate byline authors in politics (59% male and 41% female), finance (63% male and 37% women), and sport (82% males and 18% women). This lack of female representation has a significant impact on the way in which news is covered and meaning is made.

### Language and Perceptions of (in)Security

**Figure 4.**
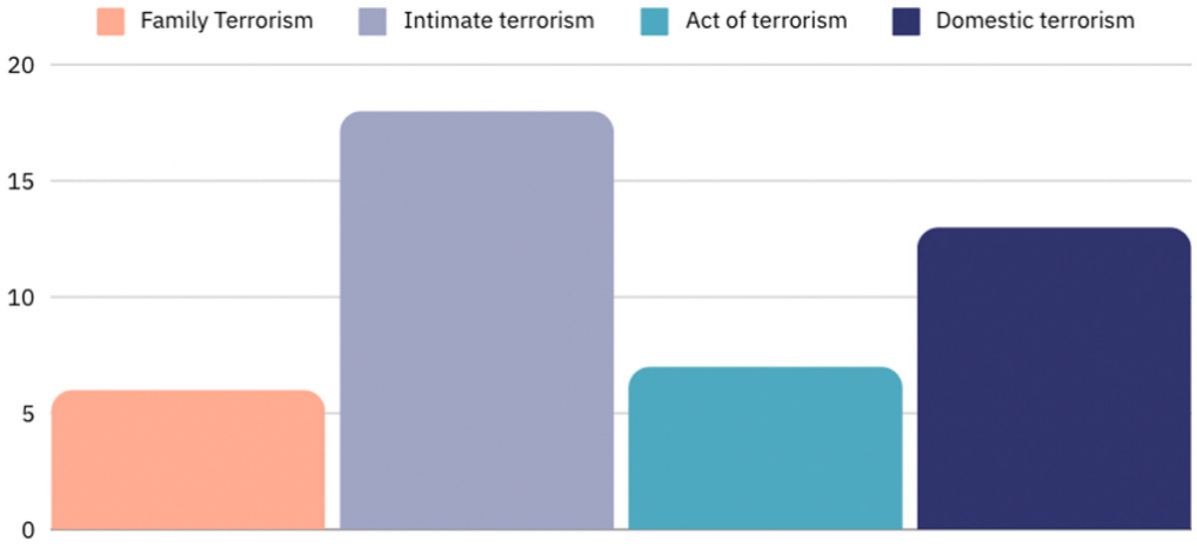
Media coverage of “terrorism” and “domestic violence” by language.

This research is also interested in when or if DFV is ever described as terrorism. Searching through the data set (of articles containing both the term “terrorism” and the phrase “domestic violence”) for instances where this language/description was used, this research found that only six articles (1.8%) used the term “family terrorism” to describe instances of DFV, 18 articles (5.3%) used the term “intimate terrorism,” seven articles (2%) described DFV as an “act of terrorism” or “terrorism,” and 13 (4%) used the term “domestic terrorism” (see Figure 4). This implies that a small minority are experimenting with this language, meaning it is certainly possible, particularly when it is coming from informed, expert voices.

A variety of individuals have made these claims: Leading the charge was Rosie Batty (23%). The influential work of Batty, in particular during this period, has been significant. Others engaged in this conversation, using terrorism to describe DFV, include Mark Speakman (Leader of the Opposition in the New South Wales Legislative Assembly), Richard Klimek (divorce lawyer and expert in family law), Dr Ann O’Neill (DFV victim-survivor and social justice researcher who lost her two children to DFV), Anne Summers (leading Australian feminist, writer, columnist and former *First Assistant Secretary of the Office of the Status of Women* in the *Department of Prime Minister and Cabinet*), Alistair Nicholson (retired *Chief Justice* of the *Family Court of Australia*), Angela Lynch (CEO of *Women's Legal Service, Queensland*), Senthorun Raj (Associate Professor in human rights law, human rights activist, and board member of *Amnesty International*), Fiona McCormack (Victoria's Victims of Crimes Commissioner), and journalists, among others. The breadth of voices that speak to this issue is a testament to the validity of the sentiment that is evidently emerging within this space.

### Data and Contextualization: Severity and Impact

The focus of this research is contextualization of (in)security, in this case context regarding the severity and impact of the issue. Regarding DFV, the inclusion or exclusion of DFV data or statistics affects the way the issue is contextualized by audiences and influences public perception of the severity, prominence, and impact of the issue. This research found that only 16.6% of articles contained data concerning DFV.

This contextualization, or lack thereof, through the inclusion or exclusion of data, is of particular interest in the coverage of DFV as coverage is most often “incident based” and governed by news values and news reporting norms. In other words, the cases that are the most violent, shocking, or newsworthy will get the most coverage. This is concerning because we know that DFV takes many forms, including financial abuse, emotional abuse, and sexual abuse (Our Watch, 2024), and these cases rarely make it into media coverage. Researchers have found that this contextualization, or lack thereof, has a significant impact on how audiences understand the nature, complexity, or diversity of the issue (Greer, 2007; Wheildon et al., 2022) impacting how DFV is discussed, understood, and inevitably prioritized or trivialized by governments and the news media. As previously explained, news coverage impacts public opinion, which impacts voting behavior.

### Access to Resources: Support in Trauma Reporting

The next data set we analyzed was access to a DFV resource. Only 2.6% of articles were found to provide access to a helpline/DFV resource somewhere in the article. This research argues that providing access to support numbers or resources is important when discussing sensitive or potentially triggering content, such as DFV. Sullivan (2018) found that “social support is critical to the wellbeing of all adults and children and is especially important for DV survivors” (p. 126). This research reflects upon this notion and advocates for a more trauma-informed approach (Simpson & Coté, 2006) that reflects upon the effect of violence on an audience, in particular DFV. Within this data set, it was found that *The Sydney Morning Herald* provided these resources considerably more than any other outlet (55.5%).

### Points of Connection: DFV and Terrorism

A core focus of this research is understanding how the conversation pertaining to DFV and terrorism plays out in news media, with the primary objective of identifying why these issues are being spoken about together and, if they are, how they are being discussed in relation to one another. This research found that 33% of all articles explicitly connected DFV and terrorism. “Explicit points of connection” in this research refers to connections being made by the news media, rather than connections made by the researcher.

The areas were connected often through the lens of funding, with journalists, interviewees, and advocates showcasing concern for the discrepancies between funding for counter-terrorism and funding for DFV. The direct comparison between these two seemingly disconnected issues is of note because it articulates reservations related to the level of concern allocated for certain issues, compared to others, by governments.

A 2015 article published by *The Sydney Morning Herald* titled “Time for Tony Abbott to step up and tackle domestic violence in Australia” lamented this perceived concern:

Domestic violence and terrorism are increasingly intertwined in the public debate, particularly by people angry that domestic violence receives, in comparison, so little funding and public attention. This, despite the fact it kills more Australians and is far more widespread than the knifing of someone in the name of Allah or the taking of hostages in Martin Place. (Metcalfe, 2015)

In a 2015 article published by the ABC titled “Budget 2015: Australian of the Year Rosie Batty says more money needed to combat ‘family terrorism’” the sentiment is again raised by Rosie Batty:

Let's put it in its context, this [DFV] is terrorism in Australia. If we look at the money that we spend in terrorism overseas, for the slight risk that it poses to our society, it is disproportionate completely. Let's start talking about family terrorism. Maybe then, with that context and that kind of language we will start to get a real sense of urgency. (ABC, 2015)

` Another key area of connection was the death tolls of each issue. The conversation pivoted on the number of deaths caused by DFV compared to terrorism and assessed subsequent government and social responses. In a 2016 article published by the ABC titled “Findings of Australia's first Royal Commission into family violence to be revealed,” the discussion pertaining to funding is again raised, but regarding the disproportionate death tolls:

We are seeing billions invested into terrorism, as we should, but there were 79 Australian women murdered on home soil…through violent means just last year alone. We need to see governments across Australia understand the importance of this. (ABC, 2016)

The areas intersect again through the lens of security or potential threats. This notion speaks to public awareness of real, emerging, and impactful threats (such as the death of women in DFV situations) compared to perceived threats such as terrorism. The issue of DFV needs immediate action to prevent further deaths and abuse, which requires state action and funding. This research has found that this funding and the introduction of public policy is often focused on counter-terrorism measures rather than measures to reduce DFV. A 2014 article published by the ABC titled “Counter-terror laws pass through Parliament with bipartisan support*”* spoke again to this notion, where the journalist posed the question:

Today you said that the paramount duty of any government is to keep our people safe; in the last decade between 700 and 1,000 Australians, nearly all of them women and children, have died at the hands of their partners and their parents. Where's the extra half billion dollars in funding to address that? Where are the additional powers for law enforcement agencies? Where's the rhetoric about existential threats for something that actually does kill Australians rather than merely threaten to kill them? (ABC, 2014)

The notion of escalation regarding private violence (that being DFV) being a precursor to public violence (that being terrorism) was also evident in this data set. As previously explained, this was seen in coverage pertaining to Man Haron Monis, the perpetrator of the Lindt Café siege, because he had previous DFV offences, and his violence escalated to terrorist acts. Alongside this type of coverage, this research found conversations pertaining to violence against women generally and governments and societies absence of urgency and action.

The inevitable intersecting areas of feminism and misogyny within discussions pertaining to DFV and terrorism saw this area as a point of connection. Conversations pertaining to the Australian government and Australian societies’ responses to violence against women being inefficient and the violence itself being undervalued is noted, in particular when compared to terrorism or acts of terror.

In a 2015 article published by *The Sydney Morning Herald* titled “Our leaders need to protect women from domestic violence” the issue of disproportionate response by governments is again raised:

In a January statement on the killing of a Japanese hostage by the “Daesh death cult,” Prime Minister Tony Abbott said it was “an absolute atrocity - an absolute atrocity.” When a second hostage was killed, he released another statement describing it as “a despicable act of terrorism.” Yet when women are brutally murdered and terrorised each week in Australia, there are no press releases, there is no urgency, no funding increase for front-line services – there is rarely any acknowledgement. (Metcalfe, 2015)

Another 2020 article published by *The Sydney Morning Herald* in their Letters to the Editor titled “Building a case against Sydneys so-called ‘progress'” spoke to the disproportionate response towards DFV (compared to terrorism):

In response to the violence perpetrated by some men on women, it is being said that: “If this was a terrorist attack there would be an immediate response”. These attacks are terrorist attacks. Often more violent, persistent, relentless, intimidating, harrowing and terrifying than political terrorism. Violence against women is allowed to develop into terror and murder and should be so described. (The Sydney Morning Herald, 2020)

## Discussion

### Contextualizing DFV: Perceptions of Severity and Impact

According to Smith (2019), DFV is much more common and far more dangerous than people perceive it to be. This notion is useful in understanding the impact and influence of news media coverage of the issue on public perceptions of severity and impact. This research argues that contextualizing DFV requires the inclusion of data to assist in painting an accurate picture of the nature of the issue in Australia, particularly when considering the case-based nature of media coverage of DFV (Robinson & Yoshida, 2022), victims under reporting instances of DFV (AIHW, 2024), and the diversity of abuse, including coercive control. This research found that DFV data that contextualized the issue (rather than simply covering each case individually and disregarding the rates and impact of DFV) was rarely included in stories that mention DFV. This is problematic and contributes to an ill-informed population that is unable to grade the severity of DFV accurately and understands DFV as a series of isolated incidents, rather than a social issue.

This research is concerned with the fact that DFV is often only reported on when it includes murder or extreme acts of violence: These cases then provide Australians with an image of what DFV looks like in this country. This research argues that this image, in reality, is far more diverse and far more severe than the media presents it to be. In regard to news coverage of terrorism, this paper found consistent contextualization of the issue, regarding the “spate of attacks” or “list of terrorism incidents,” and a lack of contextualization of DFV that, as a result, diminished the severity, impact, and pervasiveness of the issue. This mediated version of reality, where information is included or excluded, presents a warped image of the true reality of DFV in Australia, undermining the experiences of victims and DFV research. Again, this results in an inaccurate and uncontextualized presentation of security threats, in the case of this research, DFV. This research argues that this indicates audiences are not as informed as they can possibly be about such a pertinent issue and therefore are unable to make informed political decisions.

### Themes: Framing Violence

The national discussion pertaining to DFV and terrorism is heavily influenced by significant events, people, and movements. As seen in April of 2024, the conversation regarding DFV in Australia intensified, with the number of cases and the nature of these cases garnering significant attention. The conversation also took place in light of a mass stabbing at Bondi Westfield (Russel et al., 2024) and a terrorism incident (stabbing) at a Sydney church (Murphy, 2024) that received significant action and attention by governments. In the wake of these incidents, coverage again explored the connection between terrorism and DFV (Karvelas, 2024; Price & Karvelas, 2024) raising concerns about the level of consideration and action given to one form of violence (terrorism) and not the other (DFV).

This is reminiscent of the spike in coverage in 2015, as discussed earlier in this paper. The series of impactful DFV incidents, alongside an increase in funding, urgency, and action on counter-terrorism, saw Australians question the approach to these two forms of violence that threaten the security of Australians.

Exploring this national discussion, in both a modern context and within the data set enables this research to identify contexts and contributing factors that influence these patterns of public discussion and debate, where two seemingly disconnected ideas are connected. This is useful as it enables insight into how, and if, the public considers these two issues together, and if there is an ability to begin understanding DFV as a form of terrorism to expand preconceived ideas about DFV. The hope is that this re-conception can lead to impactful change and an increased level of consideration of the security threat posed by DFV.

### DFV and Terrorism: Points of Connection

The key objective of this research was to assess and understand how Australian news media frame DFV and terrorism, in conjunction with each other. The purpose of this was to explore how these two (in)security issues are being discussed and to identify how they are connected within the media coverage, either intentionally or unintentionally. Assessing and understanding the nature of the coverage of both issues enables an assessment of how Australian media covers violence and shapes perceptions of (in)security.

As explained, the two issues (DFV and terrorism) are often seen as inherently disconnected. In 2014, George Brandis, a former Liberal senator, was interviewed for an article published by the ABC titled “Counter-terror laws pass through Parliament with bipartisan support.” When asked about the discrepancies in funding terrorism compared to DFV, he stated “I think you’re foolishly conflating two completely unrelated issues.” This research argues that there are significant points of connection between the two forms of violence, and identifying these, specifically those raised by news media coverage is vital and useful to understanding (in)security and perceptions of (in)security in Australia. Identifying these points of connection also enables an assessment of how the public considers these two issues in conjunction with one another. The points of connection, often subconscious, signal a preexisting understanding (through public sentiment, policy considerations, security concerns, etc.) that these two issues are intrinsically intertwined, and there are lessons and warnings to be gained from unpacking and exploring each issue in relation to the other.

## Conclusion

This research is concerned primarily with language and its influence on perceptions of (in)security, specifically, the term terrorism and the DFV epidemic in Australia. It explored the implications of how we define a phenomenon and the effect that this definition has on the way we can understand and resolve the issue. This research explored the role of Australian news media in shaping public perceptions of (in)security, specifically in relation to DFV and terrorism. An initial excavation and analysis of DFV and terrorism literature enabled these two issues to be theorized, and a solid case for using the term terrorism to describe DFV was established. Although the literature finds this description to be accurate and credible, the term is rarely used by the public, politicians, and journalists, which this research finds to be a lost opportunity to reframe the public discussion pertaining to DFV. This research investigated the points of connection between the two key security issues in news media coverage, to gauge the subconscious and conscious association of the two issues in the coverage. The two issues were often found to be work interconnected, through the lens of funding, allocation of resources, death tolls, threats to security, experiences of victims, escalation of violence from private sphere to public sphere, and violence against women. This research also found that the two issues were often framed differently, particularly regarding their threat to the security of Australians.

This research found the severity and impact of DFV were often not articulated by news media, through lack of contextualization, lack of crucial voices (victim-survivors, advocates, researchers, experts, etc.), and the case-based nature of DFV reporting that undermines the true extent of the issue. Conversely, the issue of terrorism, or in the case of this research what was most often relating to Islamist terrorism, saw a domination of conservative voices, the absence again of crucial voices (such as Muslims, experts, and researchers), and was covered disproportionately to DFV signaling a prioritization of one issue over the other, subsequently affecting the way audiences understand the severity, impact, and pervasiveness.

The findings of this paper are significant because they identify key connections between terrorism and DFV, even if the two issues are more often perceived as “disconnected” in public and political discourse. The effect that media coverage of (in)security issues, such as DFV and terrorism, has on public perceptions of danger cannot be underestimated because it affects the way Australians understand the severity, impact, and pervasiveness of violence and (in)security threats.
